# Tumor suppressive microRNA-424 inhibits osteosarcoma cell migration and invasion via targeting fatty acid synthase

**DOI:** 10.3892/etm.2013.959

**Published:** 2013-02-18

**Authors:** XING HUA LONG, JIAN HUA MAO, AI FEN PENG, YANG ZHOU, SHAN HU HUANG, ZHI LI LIU

**Affiliations:** 1 Department of Orthopedics, First Affiliated Hospital of Nanchang University, Nanchang, Jiangxi 330006;; 2 School of Humanities, Jiangxi University of Traditional Chinese Medicine, Nanchang, Jiangxi 330006, P.R. China

**Keywords:** miR-424, metastasis, osteosarcoma, fatty acid synthase

## Abstract

Numerous studies have recently suggested that miRNAs contribute to the development of various types of human cancer as well as to their invasive and metastatic capacities. The aim of this study was to investigate the functional significance of miR-424 and to identify its possible target genes in osteosarcoma (OS) cells. Previously, inhibition of fatty acid synthase (FASN) has been shown to suppress OS cell proliferation, invasion and migration. The prediction was made using the microRNA.org and TargetScan.human6.0.database. The results showed that FASN is a promising target gene of miR-424. FASN may be a direct target of miR-424 as shown by the luciferase reporter assays. Furthermore, miR-424 expression was increased in osteosarcoma cells by transfection with has-miR-424. FASN mRNA and protein expression levels were measured by RT-PCR and western blot analysis. Cell migration and invasion was measured using Transwell migration and Transwell invasion assays. Expression levels of FASN mRNA and protein were greatly decreased in U2OS cells transfected with has-miR-424. The migration and invasion of cells was significantly decreased by the upregulation of miR-424. These findings suggested that miR-424 plays a key role in inhibiting OS cell migration and invasion through targeting FASN.

## Introduction

Osteosarcoma (OS) is one of the most common primary malignant bone tumors in childhood and adolescence. It was not until the early 1970s that the introduction of doxorubicin and methotrexate with leucovorin rescue revealed the potential to improve survival. With the advent of effective chemotherapy, the 5-year survival rate of patients treated with intensive multidrug chemotherapy and aggressive local control have been reported as 55–80% (1–3). Despite the encouraging trend towards longer survival many patients still face a dismal outcome. Numerous articles have reported that the 5-year survival rate of patients with metastatic diseases is <20% (4–6). Clearly, the impact of identifying factors that govern metastasis is significant in the management of osteosarcoma.

Fatty acid metabolic pathways play an important role in carcinogenesis (7). Fatty acid synthase (FASN) is an enzyme crucial for endogenous lipogenesis in mammals responsible for catalyzing the synthesis of long-chain fatty acids. FASN is critical to sustain the biological features of cancer cells (8). FASN is expressed at high levels in a variety of human tumors (9–13) with low levels in normal tissues. Various reports have shown that inhibiting expression of FASN suppresses cancer cell proliferation *in vitro* and *in vivo* (14–19). Recent studies revealed that FASN may contribute to cancer cell metastasis (20–22). FASN is, thus, considered a novel promising target for anticancer therapy.

miRNAs are small endogenous RNAs averaging 20 to 24 nucleotides, transcribed from non-protein-coding genes or introns, which mediate translational suppression or cleavage of their target mRNAs by binding to complementary sites in their 3′UTR (23–25). A large number of miRNAs are located inside or close to fragile chromosomal sites that are frequently lost or amplified in cancer (26). miRNAs have been characterized as oncogenes, tumor suppressors or as components of regulatory pathways critical for tumorigenesis. miRNAs play an important role in tumorigenesis and metastasis.

The aim of this study was to investigate the functional significance of miR-424 and to identify its possible target genes in osteosarcoma (OS) cells.

## Materials and methods

### Cell culture and transfection

Human OS cell line U2OS (Shanghai Cell Bank, Chinese Academy of Sciences, China) was cultured in Dulbecco’s modified Eagle’s medium (DMEM) with 10% fetal bovine serum (FBS) and incubated at 37°C in 5% CO_2_. U2OS cells were seeded in six-well plates at 30% confluence on the day before transfection. Transfection with has-miR-424 or negative miRNA was performed using Lipofectamine 2000 (Invitrogen, Carlsbad, CA, USA). Transfection complexes were prepared according to the manufacturer’s instructions. Our study was approved by the ethics committee of the First Affiliated Hospital of Nanchang University, Nanchang, China.

### Quantitative real-time PCR (qRT-PCR)

Total RNA from cells treated with has-miR-424 or negative control miRNA was isolated using TRIzol reagent (Tiangen, Beijing, China) and reverse transcribed using a reverse transcription kit (Tiangen) according to the manufacturer’s instructions. Reactions were performed and analyzed using an ABI 7300 system (Applied Biosystems, Carlsbad, CA, USA). β-actin was used as the internal control to quantify initial cellular transcripts. Details of the primers and probes used in this study are summarized in Table I. All qRT-PCR were performed six times according to the manufacturer’s instructions. The relative expression level of FASN was normalized to that of β-actin by ^2-ΔΔCt^cycle threshold method. The ΔCt data were collected automatically. The average ΔCt of each group was calculated using the following formula: ΔCt = average miR-424 Ct - Δaverage of β-actin Ct. ΔΔCt was calculated by ΔΔCt=ΔCt of miR-424 group - ΔΔCt of the negative control group. The fold-change in FASN expression level was calculated using 2^−ΔΔCt^.

### Luciferase activity assay

Primers were designed in accordance with the Genbank query FASN gene mRNA (NM_004104.4) sequence. A fragment of the 3′-UTR of FASN was amplified from U2OS cells by PCR using the forward primers 5′-CCCCTCGAGCCTGCCACCGGAGGTCACT-3′ and the reverse primers 5′-CGGGCGGCCGCGTGGGAGGC TGAGAGCAGCA-3′. After digestion of the PCR product by *Xho*I and *Not*I, the FASN 3′-UTR was cloned in pSiCHECK2 (Promega, Madison, WI, USA) at the *Xho*I and *Not*I sites. All PCR products were verified by DNA sequencing. U2OS cells were cotransfected with the pSiCHECK2 vectors containing the 3′-UTR variants and has-miR-424 or negative miR-001. Luciferase activity was measured 48 h after transfection. The firefly luciferase activity was then normalized against the renilla luciferase activity.

### Transwell invasion assay in vitro

Invasion assays were performed in triplicate using Transwell invasion chambers coated with Matrigel (50 *μ*l per filter) (BD Biosciences, Franklin Lakes, NJ, USA) as described in the manufacturer’s protocol. U2OS cells were transfected with either has-miR-424 or negative control oligonucleotide, cultured for 48 h and transferred on the top of Matrigel-coated invasion chambers in a 1% fetal calf serum DMEM/F12 (2×10^4^ cells/well). DMEM/F12 containing 10% fetal calf serum was added to the lower chambers. After incubation for 24 h at 37°C in an atmosphere containing 5% CO_2_, invaded cells on the lower surface were stained with crystal violet stain and counted under a light microscope. All experiments were repeated six times over multiple days.

### Migration assay

Migration assays were performed using a 24-well Transwell chamber system (Costar 3422, Corning Inc., NY, USA). Cells were seeded in the upper chamber at 2×10^4^ cells/ml in 0.1 ml serum-free DMEM/F12 media. Media supplemented with 10% fetal bovine serum was placed in the bottom well in a volume of 0.8 ml (used as a chemoattractant). After incubation for 24 h at 37°C in an atmosphere containing 5% CO_2_, migrated cells on the lower surface were stained with crystal violet stain and counted under a light microscope. All experiments were repeated six times over multiple days.

### Western blot analysis

U2OS cells in the exponential growth phase were transfected with has-miR-424 for 48 h. Total proteins were isolated from U2OS cells. Protein concentrations were measured using a Micro BCA protein assay kit (Pierce, Biotechnology, Inc, Rockford, IL, USA). Proteins were resolved using 10% SDS-PAGE gel, transferred to the nitrocellulose membrane, blocked in 5% non-fat dry milk in Tris-buffered saline (pH 7.4) containing 0.05% Tween-20 and blotted with a rabbit polyclonal antibody against FASN (1:1,000; Santa Cruz Biotechnology, Inc., Santa Cruz, CA, USA) and goat anti-rabbit IgG (1:3,000; Santa Cruz Biotechnology, Inc.). GAPDH was used as a loading control. Signals were detected using secondary antibodies labeled with HRP. All western blot analyses were performed six times.

### Statistical analysis

Data were expressed as means ± SD of at least six experiments. The independent samples test was used for statistical analysis. P<0.05 was considered to indicate a statistically significant result. All analyses were performed using SPSS Version 13.0 (Statistical Software for Social Sciences, Chicago, IL, USA).

## Results

### Upregulation of miR-424 inhibits cell migration in vitro

**Figure 1:**
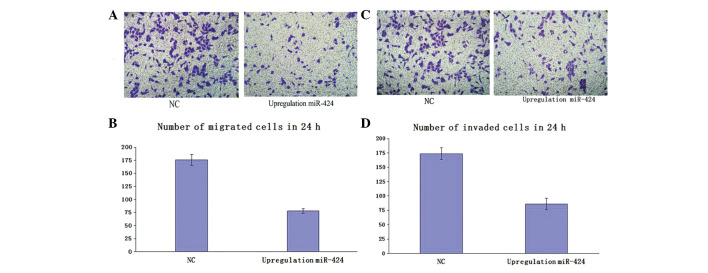
Upregulation of miR-424 suppresses osteosarcoma (OS) cell migration and invasion. (A) and (C) A representative image of six experiments of Transwell migration and invasion assays is shown for each group (10×10), respectively. (B) Columns, mean (n=6); bars, SD. ^*^P<0.05, vs. negative control group (NC). It indicated that upregulation of miR-424 expression in the U2OS cells suppressed cell migration. (D) Quantification of cell invasion expressed by cell counting. Columns, mean (n=6); bars, SD. ^*^P<0.05, vs. negative control group (NC). It indicated that upregulation of miR-424 significantly inhibits cell invasion.

To corroborate the effect of the upregulation of miR-424 on U2OS cell migration, migration was measured by Transwell migration assay. U2OS cells were transfected with miR-424 or negative miRNA. The results showed that the migration of cells transfected with has-miR-424 was significantly inhibited when compared with cells transfected with negative miRNA (Fig. 1A and B, P<0.05). These results suggested that upregulation of miR-424 inhibited the migration of U2OS cells.

### Upregulation of miR-424 inhibits cell invasion in vitro

To examine the effect of upregulation of miR-424 on U2OS cell migration, the Transwell invasion assay was performed. U2OS was transfected with has-miR-424 or negative miRNA. The results showed that the invasion of cells transfected with has-miR-424 was significantly inhibited when compared with cells transfected with negative miRNA. (Fig 1C and D; P<0.05). These results suggested that upregulation of miR-424 inhibits the invasion of U2OS cells.

### FASN is a direct target of miR-424

**Figure 2:**
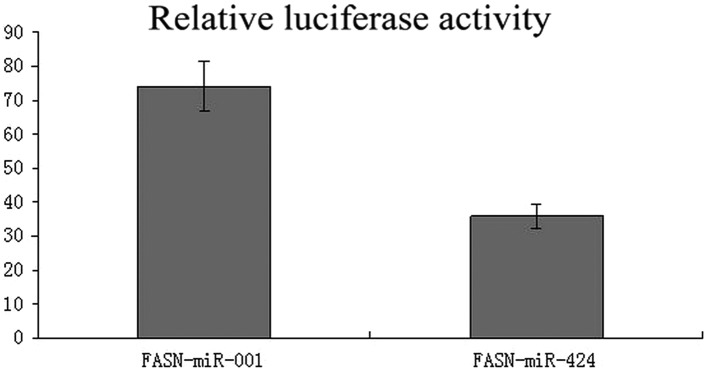
Analysis of the reporter activity in U2OS cells. The luciferase activity was normalized by renilla luciferase. Columns, mean (n=6); bars, SD. ^*^P<0.05 vs. fatty acid synthase (FASN)-miR-001 group. It indicated that miR-424 inhibits reporter activity of gene expression by targeting FASN 3′-UTR in osteosarcoma (OS) cells.

To validate whether miR-424 regulates FASN directly through a putative binding site in U2OS cells, we cloned FASN 3′-UTR in the predicted miRNA binding site into the luciferase gene (pSiCHECK2; Promega). Following cotransfection with the pSiCHECK2 vectors and miR-424 or negative control miR-001, the upregulation of miR-424 in U2OS cells transfected with has-miR-424 resulted in a significant decrease in the luciferase activity of the wild-type FASN 3′-UTR (Fig. 2; P<0.05). The results indicate that FASN is a direct target of miR-424.

### miR-424 negatively regulates FASN mRNA expression in U2OS cells

To investigate the effect of upregulation of miR-424 on the expression of FASN mRNA, miR-424 was upregulated in U2OS cells by treatment with has-miR-424 for 48 h. The expression level of FASN mRNA was measured by qRT-PCR. The data (2^−ΔΔCt^=0.254±0.01157) showed that the FASN mRNA expression in cells transfected with negative control vector was four-fold that in cells transfected with has-miR-424. It indicated that miR-424 may negatively regulate the expression of FASN mRNA.

### miR-424 inhibits FASN protein expression in U2OS cells

**Figure 3:**
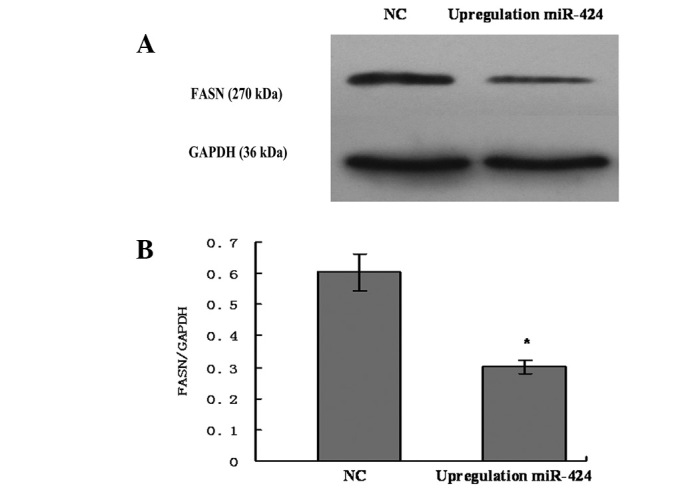
Fatty acid synthase (FASN) protein expression was inhibited by upregulation of miR-424 in U2OS cells. (A) A representative image of western blots showing the expression of FASN protein was suppressed by upregulation of miR-424. (B) Columns, mean (n=6); bars, SD. ^*^P<0.05, vs. negative control group (NC). It indicated that miR-424 inhibits FASN protein expression in U2OS cells.

To investigate the effect of upregulation miR-424 on the expression of FASN protein, miR-424 was upregulated in U2OS cells by treatment with has-miR-424 for 48 h. The expression level of FASN protein was measured using western blot analysis. The results revealed that the upregulation of miR-424 in OS cells resulted in decreasing the expression of FASN protein (Fig 3A and B). It suggested that miR-424 may negatively regulate the expression of FASN protein.

## Discussion

miR-424, one of the miR-16/15/195/424/497 family members, induces muscle differentiation and promotes cell cycle quiescence and differentiation (27–29) and regulates cell-autonomous angiogenesis (30–32). Recent evidence demonstrated that miR-424 plays an important role in tumorigenesis (33–34). miR-424 has been reported to be downregulated in cervical cancer (33), senile hemangioma (30), tongue cancer (35), chronic myelogenous leukaemia (36) and acute myeloid leukemia (34). However, miR-424 is upregulated in human colorectal cancer (37–38) and atypical chronic myeloid leukemia (37). Deregulation of miR-424 may be different in different types of cancer and the roles of miR-424 in carcinogenesis and progression should not be assumed as a tumor suppressor or oncogene. The roles of miR-424 deregulation in cancer development remains to be further investigated. In the present study, miR-424 was downregulated in the human osteosarcoma cell line U2OS. The inhibiting effect on OS cell migration and invasion by upregulation of miR-424 was observed. It suggested that miR-424 plays a role as a tumor suppressor in inhibiting OS cell migration and invasion.

A previous study reported that inhibition of FASN causes a decrease in OS cell invasion and migration. The prediction was made using software (microRNA.org and TargetScan. human6.0). It revealed that miR-424 may be targeting FASN. In this study, to investigate the molecular mechanism that inhibits the migration and invasion by restoration of miR-424 in OS cells, RT-PCR and western blot analysis were performed to detect the expression level of FASN mRNA and protein in the OS cell line U2OS. The results showed that FASN expression was significantly inhibited in cells transfected with has-miR-424 compared with the control group (Fig. 4). This suggested that restoration of miR-424 expression could inhibit FASN expression in OS cells. Furthermore, to identify whether miR-424 regulates the expression of FASN, the FASN 3′-UTR was cloned into the pSiCHECK2, placing the 3′-UTR with most potential miRNA binding sites downstream of coding sequence of luciferase (pMiR-Report; Promega). OS cells were cotransfected with the pSiCHECK2 vector containing the 3′-UTR and miR-424 or negative miR-001. Overexpression of miR-424 significantly reduced luciferase activity from the reporter construct containing the FASN 3′-UTR. It indicated that FASN is a direct miR-424 target. There are hundreds of predicted targets of miR-424 in the TargetScan prediction and a single miRNA is demonstrated to target multiple mRNAs to regulate gene expression (39), therefore it is probable that other targets of miR-424 may also participate in OS migration and invasion. miR-424 may also target different molecules in different types of cancer. Additionally, the tumor micro-environment may influence tumor progression, invasion and migration. This study indicates that expression of FASN is negatively regulated by miR-424 through a special binding site of the FASN 3′-UTR. Moreover, miR-424 inhibits cell invasion and migration in OS cells *in vitro*. These results suggest that miR-424 plays a key role in inhibiting OS cell migration and invasion by targeting FASN. Further research is necessary to identify the entire roles of miR-424 in osteosarcoma metastasis.

## Figures and Tables

**Table I:** Primers and probes used.

Primers and probes	Sequences 5′-3′
FASN	
Sense	5′-AAGCAGGCACACACGATGG-3′
Antisense	5′-TCGGAGTGAATCTGGGTTGATG-3′
Probe	5′-CTGCGGCTGCTGCTGGAAGTCACC-3′
β-actin	
Sense	5′-TGCCCATCTACGAGGGGTATG-3′
Antisense	5′-CTCCTTAATGTCACGCACGATTTC-3′
Probe	5′-CCTGCGTCTGGACCTGGCTGGC-3′

FASN, fatty acid synthase.
